# Clinical teachers’ perceptions of role modeling: a qualitative study

**DOI:** 10.1186/s12909-021-02648-1

**Published:** 2021-05-06

**Authors:** Elaheh Mohammadi, Azim Mirzazadeh, Hooman Shahsavari, Amir Ali Sohrabpour

**Affiliations:** 1 Education Development Center, Tehran University of Medical Sciences, Tehran, Iran; 2 Health Professions Education Research Center, Tehran University of Medical Sciences, Tehran, Iran; 3 Department of Medical Education, School of Medicine, Tehran University of Medical Sciences, Tehran, Iran; 4 Department of Internal Medicine, Tehran University of Medical Sciences, Tehran, Iran; 5 Medical-Surgical Department, School of Nursing and Midwifery, Tehran University of Medical Sciences, Tehran, Iran; 6 Liver and Pancreatobiliary Diseases Research Center, Digestive Disease Research Institute, Tehran University of Medical Sciences, Tehran, Iran

**Keywords:** Medical education, Clinical educator, Role model, Faculty development program, Perception, Qualitative exploration

## Abstract

**Background:**

Role modeling has been significantly considered in medical education in recent decades. In the clinical course, students learn necessary skills and accordingly their professional identity is formed by observing and working among clinical educators. Given the importance of the role modeling in medical education, in the present study, it was attempted to explore the clinical teachers’ perceptions of being a role model for medical students using a qualitative method.

**Methods:**

A qualitative design, based on the content analysis approach, was used to analyze the perspectives of 15 clinical teachers. Participants were chosen by purposeful sampling. Data were collected using reflection paper writing.

**Results:**

During the data analysis, five main categories emerged: *influencing others*, *developing different dimensions of student*, *situational self-awareness*, *feedback* and *continuous effort*.

**Conclusions:**

This study will be useful to form role modeling educational programs. Encouraging clinical teachers to make continuous efforts to improve role modeling and educating time management and self-control skills can help reduce the challenges of role modeling for clinical teachers.

## Background

Nowadays, role modeling is greatly accepted in medical education and applied as an educational tool in it. The role modeling significantly influences medical students’ values, attitudes, ethics, and professional behavior and plays a major role in choosing their future careers [1–3]. A role model is a person whose behavior or success can be emulated by others [4]. According to Bandura’s social learning theory, learning from role models is described as observational learning. According to this theory, effective learning occurs by observing others’ actions and outcomes of their behaviors [1]. Since a good role model plays a significant role in training a good physician, it is required to take a holistic view on this topic and also to gain more knowledge about clinical teachers’ perceptions of themselves as role models [5, 6]. Exploring clinical teachers’ perceptions of role modeling can provide valuable information about role modeling behavior to other clinical educators [7]. Also, this information can be applied to train them and plan faculty development programs [8–10]. Thus, conducting research on this can be helpful to foster role modeling [11].

Most studies on role modeling have investigated the role modeling from the perspective of students or the ways through which they are influenced by role models [9, 11–16]. A number of studies have also explored faculty members’ viewpoints of themselves as role models by quantitative questionnaire-based designs [8, 17]. Although valuable information can be obtained from such studies, the clinical teachers’ perceptions of role modeling have been not deeply explored in them. Another group of studies has specifically investigated the characteristics of a good role model from the perspective of role models themselves [8, 11, 18]. A study has investigated faculty members’ perceptions of role modeling after attending a training course [4]. It seems that in such a study, clinical teachers’ perceptions of role modeling was somewhat influenced by the educational intervention. Thus, clinical teachers’ initial and non-manipulated perceptions of role modeling is still hidden for us and has been not comprehensively explored in previous studies, and in the present study, it was attempted to clarify this.

## Methods

### Aim

The present study aimed to explore clinical teachers’ perceptions of being role models for medical students.

### Design

A qualitative design using conventional content analysis was conducted for perception exploration about role modeling. A qualitative content analysis method allows the deep exploration of experience, as well as interpretation of the data, leading to conclusions about the meaning of these experiences [19, 20].

### Setting

Learning through role modeling is one of the key characteristics of workplace learning and has been often considered in medical education [3, 9, 21]. Role modeling is rarely used as part of educational programs of faculty members in Iran. Learners implicitly learn from role models by observing their behaviors and their outcomes.

### Participants

The population included the clinical faculty members of different specialties of two major hospitals affiliated to Tehran University of Medical Sciences. From those eligible for inclusion, 15 (9 female) clinical teachers were purposefully selected. The inclusion criteria were being responsible for supervising residents, interns, or clerkship students in the hospital while providing clinical services as well as previous participation in development programs on reflective education. None of the faculty members received formal teaching about role modeling.

### Data collection

To collect data in the present study, reflection papers were used to collect the clinical teachers’ experiences of being a role model. All participants were given a reflection form with three open-ended questions and they were asked to reflect on their role as a behavioral model. The questions were as follows:

1. Please describe your experience of being a role model for students in the clinical setting. (What does role modeling mean to you as a clinical educator? What is your experience as a clinical role model for students?).
2. Please analyze the status of your role modeling. (What are your positive and negative experiences of being a role model as a clinical educator? What is your perception of role modeling circumstances?)
3. What do you do to be a positive and more effective role model?

### Data analysis

Data were analyzed using the conventional qualitative content analysis method [22]. The reflection papers were read several times by the first author (EM) for better understanding and general comprehension. Then, each reflection paper was divided into meaning units. The meaning units were coded and codes were divided into sub-categories and categories based on similarities and differences.

### Rigor

Each of the analytic reports was reviewed in terms of reflecting the viewpoints of participants. The research team reflected on the findings of the study and reached a consensus. A summary of the reflection papers was returned to the participants as a member check and approved by them [22]. Peer check was also performed by two authors who were familiar with the qualitative content analysis method (HS and AM). To confirm dependability, several reflection papers were randomly analyzed by an audit to ensure the accuracy of the process. The plausibility of the findings confirmed that the analyses and interpretations were justifiable.

## Results

In the present study, participants explored their perceptions of role modeling by writing a reflection paper. The results of the qualitative content analysis can be presented in 5 categories of *influencing others*, *developing different dimensions of student*s, *situational self-awareness*, *feedback,* and *continuous effort*.

### Influencing others

According to the participants, being influential was one of the components of role modeling. They believed that they could be very influential in the role modeling, greatly influence different people, including learners, colleagues, patients, etc., and make changes in people. According to the participants, a part of role modeling was to positively and negatively influence others, especially learners.

*“For an educator, being a role model means that some students can apply the positive and negative points of his or her personality in their future lives and professions after spending a period of time with that educator. An educator who spends most of his or her time earning money from visiting patients becomes a model from whom his or her student learns to behave to patients in the same way in the future.” (Participant-2)*

### Developing different dimensions of student

A role model should help his or her students develop their different dimensions. Participants believed that role models play a major role in educating learners in various educational, ethical, and clinical areas. The various dimensions mentioned by the participants were categorized in the sub-categories of “influence on clinical skills”, “influence on metacognitive skills” and “influence on professional behavior.”

### Influence on clinical skills

According to the participants, a role model can improve students’ clinical skills. According to them, the clinical teacher’s knowledge and expertise play a major role in his or her role modeling. In clinical knowledge and practice, students consider their teachers as role models and emulate their ways of performing medical practices.

*“When I responsibly and carefully perform the treatment and diagnostic procedure for a patient, the learner learns the same and I have seen that residents do the same. For example, in the case of endoscopy, I wrote a complete endoscopy report and it made the learner do the same.” (Participant-7)*

### Influence on metacognitive skill

According to the participants’ perceptions of role modeling, the effect a role model has on learners’ metacognitive skills was emphasized. Learners learn metacognitive skills such as problem-solving, clinical decision making, situational management, and critical thinking from their models.

*“The student learns how to look around, make clinical decisions, and solve problems correctly from his or her educator. I think I was a good role model for my student, since I have tried to show them how to correctly make decisions based on their situations.” (Participant-5)*

### Influence on professional behavior

According to the results of data analysis, role models play an effective role in forming and improving professional behavior of students. They are considered by students in communication skills and professional principles.

*“I always try to establish influential emotional communication with patients and their families and encourage students to do so. I explain the disease to patients and their families in the presence of learners to more familiarize them with how to interact positively with patients.” (Participant-12)*

### Feedback

Feedback was another category considered necessary to be a role model by clinical teachers. Participants’ experiences of being role models indicated the need to get feedback from others.

*“I always ask for feedback from learners at the end of theoretical classes. I also try to ask for feedback from colleagues in the middle and end of the course. The head of the ward also gives me feedback once a week.” (Participant- 2)*

According to participants, providing feedback is also effective in being a role model.

*“It is necessary to remind the student’s scientific and behavioral mistakes in a calm and private environment to avoid the destruction of his/her personality.” (Participant-15)*

### Situational self-awareness

According to the participants’ perceptions of role modeling, awareness of one’s position and role as a model is considered as the basis of role modeling. In fact, the role model reflects his or her behaviors by knowing his or her duties, and he or she can fulfill this role better through behavior control. This category includes two sub-categories of “awareness and reflection” and “behavior control”.

### Awareness and reflection

One of the components of being a role model is the awareness of role modeling. Role models should know that their students pay attention to their behavior and actions at all times and learn from them.

*“I feel I have a heavy responsibility to teach hospital courses. Because in addition to teaching theoretical courses, professional behavior, and communication skills, I must observe them myself first of all. What makes me a role model for students is my behaviors.” (Participant-9)*

### Behavior control

Role models knew behavior control as an essential part of their roles. They believed that all their actions and behaviors are carefully observed by others, so it is necessary to control their behaviors and act cautiously and wisely. To this end, self-control skills are required.

*“I feel that students monitor the teacher’s personality, behavior, and appearance. Thus, the teacher requires to be careful in all his/her actions and communications, and any mistake in them can have unfavorable effects in student education.” (Participant-17)*

### Continuous effort

According to the participants’ perceptions of role modeling, educational teachers must endeavor continuously to become a role model. Different aspects of role modeling and working in unstable clinical settings complicate this role. On the other hand, being a role model is a function of environmental conditions. Conditions such as multiple duties of faculty members, students’ learning and enthusiasm, teacher’s individual ability, and academic rank affect the quality of role modeling. It is difficult to be a role model when taking educational, research, and therapeutic roles, so teachers require motivation and continuous effort to address it in the right manner.

*“I know that sometimes I do not perform well. I try and think, but it is difficult to treat, educate, and research together. As a model, I must be able to allocate a given time to education under the ward conditions of high workload and stress for critically ill patients, and the current situation makes this more difficult.” (Participant-3)*

## Discussion

Considering the importance of role modeling in clinical education, exploring clinical teachers’ perceptions of role modeling can be helpful to foster role modeling [11]. Currently, there is no specific study related to exploring clinical teachers’ perceptions of role modeling. Thus, we designed this study for exploring clinical teachers’ perceptions of being role models for medical students.

So far, limited studies similar to ours have addressed perceptions of clinical teachers of role modeling. In this regard, Silva et al. [8] specifically investigated the characteristics of a good role model from the perspective of clinical tutors. Clinical tutors believed that they were nominated as good role models because they demonstrated empathy towards patients, good relationships with students and enthusiasm for their profession. Our study has confirmed these findings.

Moreover, Nouri et al. in 2013 explored the perspectives of nursing instructors about role modeling. According to the findings of this study role models can develop students emotionally, spiritually, and intellectually as different dimensions of human existence or humanization [23]. Although this study was related to perspectives of nursing instructors, its results were similar to our results in the category of “developing different dimensions of student”.

Our previous study in 2020 was the most relevant study in which explored the clinical teachers’ perceptions of role modeling. Closer attention to role modeling and effort for its promotion, deliberate effort to display role modeling, and creating an environment to increase the effectiveness of role modeling were three main categories which highlighted clinical teachers’ perceptions of role modeling after participating in a role modeling educational program [4]. Some findings such as “deliberate effort to display role modeling” were not found in the present study. These differences can be due to the effect of the educational program on people’s perceptions.

According to the results of the present study, the role models’ “influence on others” was the first category extracted. Role modeling is an effective method of teaching and learning. It is vital for teachers and learners [1] and considered the strongest ways to transmit intellectual and behavioral models to students [24]. A study indicated observing role models has a greater impact on students’ learning compared to formal education [25]. Role models greatly influence students’ choice of specialty [2, 7, 26]. Khan et al. [27] considered students’ choice of career as the most important influence role models have on them. Although role models play a key role in creating positive behaviors and professional development of future physicians [27, 28], their influences are not always positive and constructive. Based on a study by Curry [29], students emulated the role models’ negative behaviors they observed in the operating room. According to another study, students who observed immoral behaviors reported more immoral acts, and no significant change was made in the report of immoral behaviors after receiving more hours of ethics training [30]. In this regard, Paice et al. [5] argued that observing immoral and wrong behaviors of clinical educators makes students confused, anxious, and angry, so, according to them, role modeling is not a reliable solution to transmitting values and professional behaviors. In their review study, Passi et al. [9] introduced role models’ negative behaviors as a major challenge for clinical educators around the world in the twenty-first century. However, some studies stated that those students having the ability to reflect and think critically can avoid repeating the observed unprofessional behaviors [31–33].

“Developing different dimensions of student” by role modeling was another category extracted in our study. According to the clinical teachers participating in this study, role models play a major role in developing students’ different abilities including clinical skills, metacognitive skills, and professional behavior. The results of the present study are consistent with a systematic review study in 2013 and a narrative review study in 2019, which introduced clinical skill, effective teaching, and personality trait as role models’ three key characteristics [21, 34]. Developing students’ clinical skills is among the primary and essential goals of medical education. Clinical teachers, as role models, are responsible for teaching clinical skills to their students, and demonstrating clinical skills is one of the most important aspects of role modeling [17]. In addition, a good role model helps students to improve their metacognitive and intellectual skills such as problem-solving ability, critical thinking, and clinical decision-making, and he/she plays a major role in their intellectual development [23, 35]. Role models play a major role in shaping students’ professional behavior and emotional development [2, 36, 37]. Hunter and Cook [33] stated that students’ professional identity is shaped by encountering desirable and undesirable role models. In a study by Silvia et al. [8], educators considered empathy with patients and having good communication skills with students as the characteristics of a role model. Positive role models are kind, honest, and patient [21, 38] and with their desirable behaviors, they can develop the professional behavior in future physicians [39–41].

Another category obtained from data analysis was “feedback”. Participants believed that receiving and providing feedback are of great importance in role modeling as far as they are necessary to be a role model. Seeking feedback and receiving others’ opinions improve and enhance role modeling [4]. Providing feedback is also effective in role modeling. Benbassat [31] believed that the effectiveness of role modeling depends on its definition. If role modeling is defined as the demonstration of skills and provision of feedback after observing students’ performance, role modeling can be considered as a vital component of clinical education.

“Situational self-awareness” was another category extracted from data analysis in the present study. According to the participated clinical teachers’ perceptions of role modeling, role models should be aware of their roles and pay more attention to the positive and negative effects of role modeling. In addition, since they are role models, it is necessary for them to reflect on their behaviors to review, correct, and control their behaviors in the presence of students. A good clinical teacher should be aware of his or her performance as a role model. Such increased awareness of modeled behavior can be used to develop faculty members’ teaching skills [3, 21]. Encouraging clinical teachers to reflect on their behaviors and actions is effective in promoting their role modeling [9, 27, 42, 43]. In a study by Wright and Carrese [11], who investigated the insights of medical role models, role models were aware of their role modeling and known as successful clinicians.

“Continuous effort” was the last category clinical teachers perceived from role modeling. According to the participants’ perceptions of role modeling, being a role model has different dimensions. An educator is a role model in the areas of clinical skills, professional ethics, education, etc. and he or she should pay attention to all these aspects. Caring for patients and educating students in crowded clinical settings simultaneously challenge clinical teachers to perform role modeling duties and tasks [44]. Lack of sufficient opportunities for education and interaction with students on the one hand, and students’ motivation and activity for learning, on the other hand, make education in clinical settings difficult and challenge clinical teachers with different abilities to satisfy their role modeling. Hence, clinical teachers must continuously try to achieve this important goal. In our previous study in 2020, we explored the clinical teachers’ perceptions of role modeling after participating in a role modeling educational program. In this study was emphasized on the provision of a holistic role modeling at all times by clinical teachers as well as the deliberate display of role modeling in various aspects. It was also highlighted that role models should display their desired behavior for more effective education, and try to build a positive learning environment to increase the influence of role modeling [4].

## Conclusions

Since role modeling has many positive and negative effects on medical students’ professional identity formation, it is of great importance to explore clinical teachers’ perceptions and awareness of role modeling to develop better faculty development programs. Based on the results of this study, in addition to emphasizing the importance of role modeling and its influence on students’ professional behavior, it is helpful to include the training of skills such as the provision of appropriate feedback and reflection in such programs. Moreover, encouraging clinical teachers to make continuous efforts to improve role modeling and teaching them time management and self-control skills are useful measures to diminish the challenges of role modeling for clinical teachers and foster role modeling in the clinical settings.

## Limitations

The impossibility of generalizability of results is one of the limitations of qualitative studies. In the present study, due to the use of a small sample size and purposeful sampling method, we should generalize the results cautiously. There was lack of maximum variation sampling in our study. In addition, because of the participants’ heavy workload, we could not use different data collection methods (such as face-to-face interview) and only, a reflection technique was used. There was also lack of triangulation method in this study. Using of triangulation method can develop a comprehensive understanding of phenomena.

## Recommendations for future studies

Future studies are recommended to apply the results of the present study to design faculty development programs to improve role modeling. In addition, to enrich the content, it is recommended to use other data collection methods such as interviews along with reflection.

## Data Availability

The data generated and analysed during the current study are available from the corresponding author on reasonable request.

## References

[1] Mann K, Steinert Y (2014). Faculty development to promote role-modeling and reflective practice. Faculty development in the health professions.

[2] Passi V, Johnson N (2016). The impact of positive doctor role modeling. Med Teach.

[3] Cruess SR, Cruess RL, Steinert Y (2008). Teaching rounds: role modelling - making the most of a powerful teaching strategy. BMJ.

[4] Mohammadi E, Mortaz Hejri S, Sohrabpour AA, Mirzazadeh A, Shahsavari H. Exploring clinical educators’ perceptions of role modeling after participating in a role modeling educational program. Med Teach. 2020:1–13. 10.1080/0142159X.2020.1849590. 10.1080/0142159X.2020.1849590 33226884

[5] Paice E, Heard S, Moss F (2002). How important are role models in making good doctors?. BMJ.

[6] Mohammadi E, Mirzazadeh A, Sohrabpour A, Shahsavari H, Yaseri M, Mortaz HS (2020). Enhancement of role modelling in clinical educators: a randomized controlled trial. Med Teach.

[7] Wright S, Wong A, Newill C (1997). The impact of role models on medical students. J Gen Intern Med.

[8] Silva L, Troncon L, Panúncio-Pinto M (2019). Perceptions of occupational therapy students and clinical tutors on the attributes of a good role model. Scand J Occup Ther.

[9] Passi V, Johnson S, Peile E, Wright S, Hafferty F, Johnson N (2013). Doctor role modelling in medical education: BEME guide no. 27. Med Teach.

[10] Salajegheh M, Gandomkar R, Mirzazadeh A, Sandars J (2020). Identification of capacity development indicators for faculty development programs: a nominal group technique study. BMC Med Educ.

[11] Wright SM, Carrese JA (2002). Excellence in role modelling: insight and perspectives from the pros. CMAJ.

[12] Burgess A, Goulston K, Oates K (2015). Role modelling of clinical tutors: a focus group study among medical students. BMC Med Educ.

[13] Haider SI, Snead DR, Bari MF (2016). Medical students’ perceptions of clinical teachers as role model. PLoS One.

[14] Lombarts KM, Heineman MJ, Arah OA (2010). Good clinical teachers likely to be specialist role models: results from a multicenter cross-sectional survey. PLoS One.

[15] Boerebach BC, Lombarts KM, Keijzer C, Heineman MJ, Arah OA (2012). The teacher, the physician and the person: how faculty’s teaching performance influences their role modelling. PLoS One.

[16] Passi V, Johnson S (2016). The hidden process of positive doctor role modelling. Med Teach.

[17] Ambrozy D, Irby D, Bowen J, Burack J, Carline J, Stritter F (1997). Role models’ perceptions of themselves and their influence on students’ specialty. Acad Med.

[18] Althouse LA, Stritter FT, Steiner BD (1999). Attitudes and approaches of influential role models in clinical education. Adv Health Sci Educ Theory Pract.

[19] Jaafari F, Delavari S, Bazrafkan L (2019). Evaluation of the geriatric curriculum implemented at Shiraz University of Medical Sciences, Iran, since 2017: a qualitative study. F1000Res.

[20] Yazdani S, Farajpour A, Shakerian S (2017). Practice-based learning and improvement (PBLI) from the perspective of Iranian medical education experts: a thematic content analysis. Iran Red Crescent Med J.

[21] Jochemsen-van der Leeuw HG, van Dijk N, van Etten-Jamaludin FS, Wieringa-de Waard M (2013). The attributes of the clinical trainer as a role model: a systematic review. Acad Med.

[22] Graneheim UH, Lundman B (2004). Qualitative content analysis in nursing research: concepts, procedures and measures to achieve trustworthiness. Nurse Educ Today.

[23] Nouri JM, Ebadi A, Alhani F, Rejeh N, Ahmadizadeh MJ (2013). Qualitative study of humanization-based nursing education focused on role modeling by instructors. Nurs Health Sci.

[24] Jochemsen-van der Leeuw HG, Wieringa-de Waard M, van Dijk N (2015). Feedback on role model behaviour: effective for clinical trainers?. Perspect Med Educ.

[25] Glicken AD, Merenstein GB (2007). Addressing the hidden curriculum: understanding educator professionalism. Med Teach.

[26] Elzubeir M, Rizk D (2001). Identifying characteristics that students, interns and residents look for in their role models. Med Educ.

[27] Khan A, Yasmeen R, Awan W, Niazi S, Malik U (2020). Role modeling in medical education and its influences on professional behaviours. Ann King Edw Med Univ.

[28] Amalba A, Abantanga F, Scherpbier A, Van Mook W (2017). Community-based education: the influence of role modeling on career choice and practice location. Med Teach.

[29] Curry S, Cortland C, Graham J (2011). Role-modelling in the operating room: medical student observations of exemplary behaviour. Med Educ.

[30] Feudtner C, Christakis D, Christakis N (1994). Do clinical clerks suffer ethical erosion? Students’ perceptions of their ethical environment and personal development. Acad Med.

[31] Benbassat J (2014). Role modeling in medical education: the importance of a reflective imitation. Acad Med.

[32] Naeimi L, Asghari F, Nedjat S, Mirzazadeh A, Abbaszadeh M, Sima A (2020). Turning unprofessional behaviors around using Holmes’ reflection approach: a randomized controlled study. J Med Ethics Hist Med.

[33] Hunter K, Cook C (2018). Role-modelling and the hidden curriculum: new graduate nurses’ professional socialisation. J Clin Nurs.

[34] Mohammadi E, Shahsavari H, Mirzazadeh A, Sohrabpour AA, Mortaz HS (2019). Improving role modeling in clinical teachers: a narrative literature review. J Adv Med Educ Prof.

[35] Balmer D, Serwint J, Ruzek S, Ludwig S, Giardino A (2007). Learning behind the scenes: perceptions and observations of role modeling in pediatric residents’ continuity experience. Ambul Pediatr.

[36] Branch WT, Hafler JP, Frankel RM, Weil AB, Gilligan MC, Litzelman DK (2017). A multi institutional longitudinal faculty development program in humanism supports the professional development of faculty teachers. Acad Med.

[37] Park J, Woodrow SI, Reznick RK, Beales J, MacRae HM (2010). Observation, reflection, and reinforcement: surgery faculty members’ and residents’ perceptions of how they learned professionalism. Acad Med.

[38] Mirhaghi A, Moonaghi HK, Sharafi S, Zeydi AE (2015). Role modeling: a precious heritage in medical education. Acta Fac Med Naiss.

[39] Mortaz Hejri S, Mohammadi E (2020). Response to: medical students’ perspectives on the ‘enhancement of role modelling in clinical educators’. Med Teach.

[40] Sternszus R, Steinert Y, Bhanji F, Andonian S, Snell LS (2017). Evaluating a novel resident role-modelling programme. Clin Teach.

[41] Pinard AM, Savard I, Cote L (2017). Role modelling: moving from implicit to explicit. Clin Teach.

[42] Kenny NP, Mann KV, MacLeod H (2003). Role modeling in physicians’ professional formation: reconsidering an essential but untapped educational strategy. Acad Med.

[43] Mirhosseini F, Mehrdad N, Bigdeli S, Peyravi H, Khoddam H (2018). Exploring the concept of scholarship of teaching and learning (SoTL): concept analysis. Med J Islam Repub Iran.

[44] Cohen M, Hickey J, Upchurch S (2009). Faculty workload calculation. Nurs Outlook.

